# Cell therapy for male sexual dysfunctions: systematic review and position statements from the European Society for Sexual Medicine

**DOI:** 10.1093/sexmed/qfad071

**Published:** 2024-02-09

**Authors:** Celeste Manfredi, Luca Boeri, Ioannis Sokolakis, Nicolò Schifano, Nikolaos Pyrgidis, Esaú Fernández-Pascual, Andrea Sansone, Borja García-Gómez, Maarten Albersen, Giovanni Corona, Javier Romero-Otero, Mikkel Fode, the ESSM Scientific Collaboration and Partnership (ESCAP)

**Affiliations:** Urology Unit, Department of Woman, Child and General and Specialized Surgery, University of Campania “Luigi Vanvitelli”, Naples 80138, Italy; Department of Urology, Rush University Medical Center, Chicago, IL60612, United States; Department of Urology, Fondazione IRCCS Ca' Granda - Ospedale Maggiore Policlinico, Milan 20122, Italy; 2nd Department of Urology, School of Medicine, Faculty of Health Sciences, Aristotle University of Thessaloniki, Thessaloniki 54124, Greece; ASST Sette Laghi-Circolo e Fondazione Macchi Hospital, Varese 21100, Italy; Department of Urology, University Hospital, LMU Munich, Munich 80336, Germany; LYX Institute of Urology, Facultad de Medicina, Universidad Francisco de Vitoria, Madrid 28223, Spain; Department of Urology, Hospital Universitario La Paz, Instituto de Investigación Universitario La Paz (IdiPAZ), Madrid 28046, Spain; Chair of Endocrinology and Medical Sexology (ENDOSEX), Department of Systems Medicine, University of Rome Tor Vergata, Rome 00133, Italy; Department of Urology, Hospital Universitario 12 Octubre, Instituto de Investigación Sanitaria Hospital 12 de Octubre (imas12), Madrid 28041, Spain; ROC Clinic, Madrid 28010, Spain; Department of Urology, HM Hospitales (Montepríncipe, Puerta del Sur, Sanchinarro), Madrid 28660, Spain; Department of Urology, University Hospitals Leuven, 3000, Belgium; Endocrinology Unit, Azienda AUSL, Bologna 40123, Italy; ROC Clinic, Madrid 28010, Spain; Department of Urology, HM Hospitales (Montepríncipe, Puerta del Sur, Sanchinarro), Madrid 28660, Spain; Department of Urology, Copenhagen University Hospital - Herlev and Gentofte Hospital, Herlev 2730, Denmark; Department of Clinical Medicine, University of Copenhagen, Copenhagen 1172, Denmark

**Keywords:** erectile dysfunction, Peyronie’s disease, regenerative medicine, sexual dysfunction, stem cells

## Abstract

**Background:**

Cell therapy (CT) is a form of regenerative medicine under investigation for the management of male sexual dysfunction (MSD).

**Aim:**

We sought to perform a systematic review of published information on CT for MSD and provide an official position statements for the European Society for Sexual Medicine.

**Methods:**

A comprehensive bibliographic search on the MEDLINE, Web of Science, Scopus, and Cochrane Library databases was conducted in February 2023. Articles were selected based on the Population, Intervention, Comparator, Outcome, Study design (PICOS) model if they included male patients (P) undergoing CT (I) with or without comparison with other treatments (C) and evaluated the impact of CT on sexual function (O). Quantitative data were reported as found in the original studies (S). Level of evidence and grade of recommendation according to the Oxford Centre for Evidence-Based Medicine were assigned to each statement.

**Outcomes:**

Outcomes were determined based on assessment of erectile function, ejaculatory function, orgasmic function, sexual desire, and penile curvature.

**Results:**

A total of 19 studies and 421 patients were included. Most articles (n = 12, 63%) were case series, whereas a minority of papers (n = 6, 32%) had a comparative group; only 2 articles reported randomized controlled trials (RCTs) and 1 article reported a post hoc analysis of RCTs. Most articles (16, 84%) investigated patients with erectile dysfunction (ED). Improvements in the International Index of Erectile Function–Erectile Function Domain (IIEF-EF) or the IIEF 5-item version (IIEF-5) were found in 11/15 (73%) studies, with mean increases in IIEF-EF, mean IIEF-5, and median IIEF-EF between 8 and 14 points, 2 and 9 points, and 4.5 and 6 points, respectively. Two papers (20%) evaluated men with Peyronie’s disease (PD). In both ot these articles penile curvature improvement and plaque volume reduction were described in all patients (n = 16, 100%). Objective measurements were performed in 1 study, which showed 10°-120° (15%-100%) curvature improvement and 90%-100% plaque reduction. Mild transient adverse events at the donor or administration sites were found in 7/16 (44%) papers on ED. Priapism was reported in one case (20%) and mild penile skin complications were reported in the majority of patients after CT for PD. No severe adverse events were described.

**Clinical Implications:**

Although high-quality evidence is lacking, CT appears to have potential benefits from application in patients with ED or PD.

**Strengths and Limitations:**

This report is to our knowledge the most comprehensive and up-to-date systematic review on the topic of CT for the management of MSD, including the position statements of the European Society for Sexual Medicine. Overall the assessment of available studies demonstrated low quality and significant heterogeneity.

**Conclusion:**

Preliminary findings support potential efficacy and safety of CT in patients with ED or PD. Low-quality papers, high methodological heterogeneity, uncertainty about the magnitude of the beneficial effects, and lack of long-term data limit the available evidence.

## Introduction

Male sexual dysfunctions (MSDs) can be classified as dysfunction in sexual interest/desire, sexual arousal (ie, erectile dysfunction [ED]), premature ejaculation, orgasmic dysfunctions, and other conditions (eg, Peyronie’s disease [PD]).^1^ These MSDs can have a profoundly negative impact on couple’s fitness and a dramatic effect on quality of life.^2^

The treatment of some of these conditions has significantly improved in recent decades thanks to the combination of a psychological approach and the development of new drugs, novel surgical techniques, and innovative technologies.^1-5^ However, in recent years we have been experiencing stagnation in the available therapeutic arsenal.^6^ Furthermore, the demand for “curative” and “definitive” treatments has always been a priority for patients suffering from MSD.^7^ Regenerative medicine, which is based on treatments that promote the replacement or regeneration of damaged cells, tissues, or organs to restore normal function, has been emerging into this scientific and cultural context.^8^ This approach appears to be an interesting therapeutic option and a potential game changer in the management of patients with MSD.^6^

Cell therapy (CT) refers to the transfer of cellular material into a patient for medical purposes. It includes stem cell– and non–stem cell–based therapies, covering multiple therapeutic areas, such as regenerative medicine, immunotherapy, and antineoplastic treatment.^9^ However, it is essential to underline that in the literature “stem cell therapy” often refers to therapies based on multicellular products containing multiple stem cells and non–stem cells obtained by extraction and processing of various tissues (eg, stromal vascular fraction, bone marrow aspirate).^9^^,^^10^ The key characteristics of stem cell are the ability to self-renew and the potential to differentiate into mature cell types. Based on the differentiation potential, these cells are classified as totipotent, pluripotent, multipotent, or unipotent, and, depending on the origin, they are distinguished in syngeneic, autologous, allogeneic, or xenogeneic cells.^11^ Mesenchymal stem cells (MSCs) are among the most frequently studied cell types for regenerative medicine. They are multipotent adult stem cells that can be isolated from different tissues, including bone marrow, adipose tissue, placenta, and umbilical cord.^12^ These MSCs exert their effect in therapeutic settings through several mechanisms of action. Differentiation and replacement of damaged cells is only one of many possible mechanisms and appears to be less relevant than the other possible roles of MSCs in the tissue repair process. Cell fusion, secretion of paracrine factors (eg, growth factors, cytokines, hormones), transfer of organelles (eg, mitochondria) or molecules through tunneling nanotubes, and transfer of signals via extracellular vesicles (eg, exosomes, microvesicles) are further demonstrated mechanisms of action of MSCs.^13^ In addition to the repairing effect, MSCs have shown anti-inflammatory, immunomodulatory, angiogenic, antiapoptotic, mitotic, antifibrotic, and antioxidant properties.^14^

Several preclinical studies have explored the molecular and cellular mechanisms underlying MSC treatments and reported encouraging results on the possible use of stem cells in MSD.^10^^,^^15^ The first clinical trial on the topic, published in 2010 by Bahk et al, showed promising preliminary findings^16^; however, few studies on humans have been conducted since then. Even today, this topic is the subject of great debate due to high costs, uncertainty about efficacy, and doubts regarding safety.^10^ Moreover, no specific recommendation on CT is available in the current European Association of Urology Guidelines on Sexual and Reproductive Health,^17^ whereas according to the latest American Urological Association Guidelines, stem cells should be considered an investigational method in men with ED (conditional recommendation; evidence level: grade C).^18^

The aim of the investigation reported here was to perform a systematic analysis of the current evidence regarding CT for MSD in humans and to provide position statements for their clinical use on behalf of the European Society for Sexual Medicine (ESSM).

## Materials and methods

### General methodology

The protocol for this study was registered in the International PROSPERO (Prospective Register of Systematic Reviews) database. The data were reported according to the PRISMA (Preferred Reporting Items for Systematic Review and Meta-analysis) statement.^19^

### Search strategy

A comprehensive bibliographic search on the MEDLINE, Web of Science, Scopus, and Cochrane Library databases^20^ was conducted in February 2023 to identify relevant studies. Different combinations of the following keywords were used to search for articles by title/abstract: “stem cell”, “mesenchymal”, “regenerative”, “regeneration”, “stromal vascular fraction”, “bone marrow”, “lipoaspirate”, “sex”, “sexual”, “intercourse”, “penis”, “penile”, “testicles”, “testis”, “testicular”, “erectile”, “erection”, “impotence” “Peyronie”, “curvature”, “induratio”, “recurvatum”, “ejaculation”, “ejaculatory”, “orgasm”, “desire”, “libido”. In addition, different associations of the following MeSH (Medical Subject Headings) terms were used to search for other relevant articles that may have escaped the previous search on the MEDLINE and Cochrane Library databases: “Stem Cells”, “Coitus”, “Erectile Dysfunction”, “Penile Induration”, “Premature Ejaculation”, “Orgasm”, “Libido”, “Sexual Dysfunction, Physiological”. The literature search was limited to English language publications and studies in humans. No restrictions for the date of publication were applied (**Supplementary Data**). References lists of the retrieved articles were used to identify additional significant studies. A further literature search based on the same parameters but restricted to the last 6 months before study completion was performed before submission to detect any new relevant papers published.

### Study selection

The Population, Intervention, Comparator, Outcome, Study design (PICOS) model^21^ was applied to define study eligibility. Articles were selected if they included male patients (P) undergoing CT (I) with or without comparison with other treatments (C), evaluating its impact on sexual function (O). Prospective and retrospective original studies were included (controlled and uncontrolled, randomized and nonrandomized). Given the presumed paucity of available papers, case reports, small case series (<10 cases), and post hoc analyses were also included. Conference abstracts, reviews, comments, letters to editors without original data, animal studies, and in vitro studies were excluded (S).

With the term “CT” we meant any treatment based on substances whose effects were presumed to derive mainly from the cells contained in them or their products. Platelet-rich plasma treatment was excluded as it was considered an acellular therapy.^9^ Eligibility based on the assessment of the impact of CT on sexual function was defined as the description of erectile function, ejaculatory function, orgasmic function, sexual desire, or penile curvature using any type of validated or nonvalidated tool. Male fertility was excluded as an outcome because it falls within the reproductive rather than strictly sexual function. Articles evaluating the impact of stem cell transplantation for hematologic diseases on sexual function were excluded. Papers in which CT was part of combined treatment were included if a control arm allowed the effects of CT to be discerned. Studies with longer follow-up were chosen over articles with the same population and shorter follow-up; however, any missing data in the included articles were obtained from studies with shorter follow-up, if available.

The identification of relevant studies was conducted independently by 6 of the authors (I.S., N.P., E.F.-P., A.S., L.B., N.S.). An initial screening of titles and abstracts was performed. When it was not clear from the abstract whether the document might contain relevant data, the full article was evaluated. Thereafter, selected studies underwent a thorough full-text assessment to determine whether they were eligible for inclusion. Four senior authors (B.G.-G., J.R.-O., M.A., M.F.) supervised and resolved disagreements. No software or artificial intelligence was used in the search and selection of the articles. The bibliographies of the included studies were analyzed to find any additional relevant articles.

### Data extraction and quality assessment

The following items were recorded: first author, publication year, country of origin, study period, study design, number of patients, age of patients, follow-up, clinical setting, type of CT, treatment protocol, efficacy outcomes, and safety outcomes.

The level of evidence (LoE) of all studies was evaluated according to the instructions provided by the Oxford Centre for Evidence-Based Medicine 2011,^22^ ranging from 1 to 5 in decreasing order of evidence. The quality of the randomized RCTs, comparative nonrandomized studies, noncomparative studies, and case reports was determined with the Jadad scale,^23^ Newcastle-Ottawa scale (NOS),^24^ adapted NOS (without “selection of the nonexposed cohort” and “comparability of cohorts on the basis of the design or analysis” items),^25^ and Murad scale,^26^ respectively. Different cutoffs were arbitrarily chosen to classify the quality of the studies into low, intermediate, or high based on the scores obtained with these scales. A total score of 0-5 was considered low quality, 6 intermediate quality, and 7-9 high quality for the comparative nonrandomized studies. A total score of 0-2 was considered low quality, 3 intermediate quality, and 4-5 high quality for the RCTs. A total score of 0-4 was considered low quality, 5 intermediate quality, and 6-8 high quality for case reports. Finally, a total score of 0-3 was considered low quality, 4 intermediate quality, and 5-6 high quality for noncomparative studies.

### Data synthesis and position statements

As a relatively low number of relevant papers with high heterogeneity in methodology and poor quality were expected, quantitative data were reported as found in the original studies. Sums, percentages, and means were used to summarize the quantitative data. The characteristics and main findings of all included articles were also reported narratively.

Position statements were formulated and approved by all authors following a discussion based on the available literature and the knowledge, and clinical experience of the authors. An LoE (range 1-5) and grade of recommendation (range A-D) according to the Oxford Centre for Evidence-Based Medicine were assigned to each position statement.^22^^,^^27^ When the statement was derived from common sense rather than from study results, LoE and grade were replaced with a “Good Clinical Practice Statement.” The terms “should” and “may” were used when the statement constituted a strong recommendation or suggestion, respectively.

## Results

### ESSM position statements

1. Cell therapy for MSD should be considered a treatment under investigation and not offered outside of clinical trials approved by an Ethics Committee. (Good Clinical Practice Statement)

2. Patients should be informed regarding the limited evidence on the efficacy and safety of CT for MSD. Possible benefits, observed effects size, presumable timing and duration of effects, and potential adverse effects should be discussed in detail to set realistic expectations. (Level 3, Grade C)

3. Patients should be informed that CT for ED has been associated with improvements in erectile function and penile rigidity, but the clinical significance of the observed effects size is uncertain and the supporting evidence is limited. (Level 3, Grade C)

### Evidence

#### Main characteristics of studies

**Figure 1:**
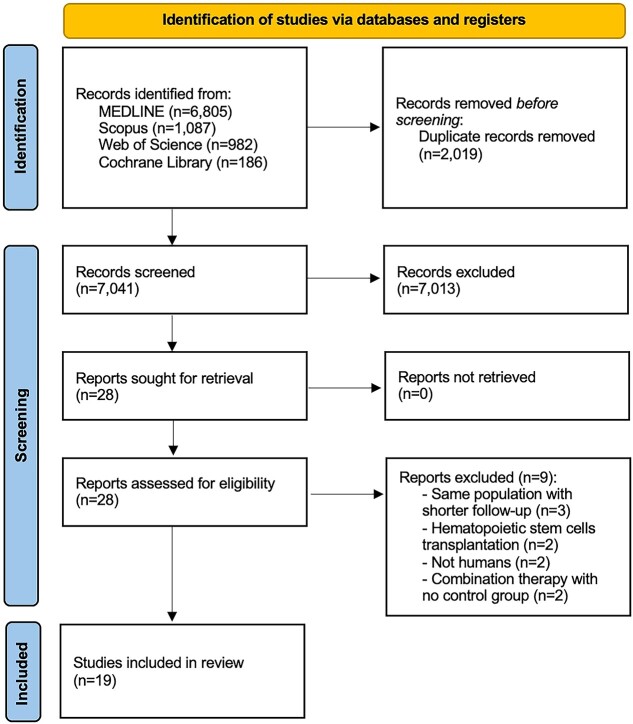
PRISMA flow diagram for study selection.

A total of 19 studies^16^^,^^28-45^ were included in our analysis (Figure 1). An overall cohort of 421 patients (median, 11; range, 1-140) was evaluated in selected articles. The follow-up ranged from 3 to 62 months, but only 2 studies (11%) presented data beyond 12 months.^33^^,^^35^ The first paper on the topic was conducted in South Korea and published in 2010.^16^ The majority of articles analyzed (n = 12, 63%) were case series ^29-35^^,^^41-45^; a minority of papers (n = 6, 32%) had a comparative group.^16^^,^^36-40^ Only 2 RCTs and 1 post hoc analysis of RCTs were identified.^16^^,^^37^^,^^40^ The main characteristics of the studies are detailed in Table 1. The countries of origin, years of publication, and designs of the included studies are graphically summarized in Supplementary Figure 1.

**Table 1:** Main characteristics of studies.

**First author and publication year**	**Country of origin** ^a^	**Study design**	**Study period**	**No. (type) of patients**	**Patient age, y (type)**	**Follow-up,** **mo** ^b^
Bahk 2010^16^	South Korea	RCT, single-blind	NA	10 (total) 7 (CT) 3 (control group)	69.5^c^	11
Ichim 2013^28^	USA	Case report	NA	1	35	18
Garber 2015^29^	Spain	Prospective, single-arm	NA	6	63.7^c^	12
Levy 2015^30^	USA	Prospective, single-arm	2013-2015	5	45-59^d^	6
Levy 2016^31^	USA	Prospective, single-arm	NA	8	40-70^d^	6
Lander 2016^32^	USA	Prospective, single-arm	NA	11	61^c^	6
Yiou 2017^33^	France	Prospective, single-arm	NA	18 (total) 6 (stage II) 12 (stage I)	59.9^c^ (stage II) 63.9^c^ (stage I)	6 (stage II) mean 62.1 (stage I)
Haahr 2018^34^	Denmark	Prospective, single-arm	2014-2015	21	60.2^c^	12
Al Demour 2018^35^	Jordan	Prospective, single-arm	NA	4	49-60^d^	24 (safety) 12 (efficacy)
Protogerou 2019^36^	Greece	Prospective, 2-arm, nonrandomized	NA	8: 5 (CT + platelet lysate) 3 (platelet lysate)	NA	3
Ory 2020^37^	Canada	Post hoc analysis of 3 RCTs	NA	36: 8 placebo 28 CT	65^c^ (placebo) 65^c^ (CT)	12
Zasieda 2020^38^	Ukraine	Prospective, 2-arm, nonrandomized	NA	38: 19 CT + ESWT 19 ESWT	NA	3
Bieri 2020^39^	USA	Prospective, 2-arm, nonrandomized + One-arm registry	NA	140: 40 clinical trial (20 low dose, 20 high dose) 100 registry	36^c^ (low dose), 52^c^ (high dose), 57^c^ (registry)	6
Mirzaei 2021^40^	Iran	RCT, single-blind	2019-2020	20: 10 CT 10 (control group)	63.8^c^ (CT), 65.6^c^ (control group)	6
Nguyen Thanh 2021^41^	Vietnam	Prospective, single-arm	2017-2020	15	48.4^c^	12
You 2021^42^	Korea	Prospective, single-arm	2015-2019	10	62^c^	12
Al Demour 2021^43^	Jordan	Prospective, single-arm	2018-2019	22	59.2^c^	12
Koga 2021^44^	Japan	Prospective, single-arm	2016-2020	38	56^c^	2
Fode 2023^45^	Denmark	Prospective, single-arm	2020	10	61^d^	3 (efficacy) 6 (safety)

Abbreviations: CT, cell therapy; ESWT, extracorporeal shock wave therapy; NA, not available; RCT, randomized controlled trial.

^a^First author.

^b^Last visit, unless otherwise stated.

^c^Mean.

^d^Range.

^e^Median.

LoEs of 4 and 2 were assigned to 16 (84%)^28-36^^,^^38^^,^^39^^,^^41-45^ and 3 (16%)^16^^,^^37^^,^^40^ papers, respectively. Analysis of study quality revealed a median (range) NOS score of 5 (4-5) for the comparative nonrandomized studies (overall low quality), a median (range) Jadad scale score of 3 (2-4) for the RCTs (overall intermediate quality), a median (range) adapted NOS score of 4 (3-5) for the comparative nonrandomized studies (overall intermediate quality), and a Murad score of 3 for the only case report included (low quality). The study quality and LoE assessment was detailed in Table 2.

**Table 2:** Quality and level of evidence of studies.

**Reference**	**Study quality/risk of bias, total score** ^**a**^	**Level of evidence** ^**b**^
Bahk 2010^16^	2^c^	2
Ichim 2013^28^	3^d^	4
Garber 2015^29^	4^e^	4
Levy 2015^30^	5^e^	4
Levy 2016^31^	4^d^	4
Lander 2016^32^	3^d^	4
Yiou 2017^33^	5^d^	4
Haahr 2018^34^	4^d^	4
Al Demour 2018^35^	3^d^	4
Protogerou 2019^36^	4^c^	4
Ory 2020^37^	3^e,f^	2
Zasieda 2020^38^	5^c^	4
Bieri 2020^39^	5^c^	4
Mirzaei 2021^40^	4^e^	2
Nguyen Thanh 2021^41^	4^d^	4
You 2021^42^	4^d^	4
Al Demour 2021^43^	4^d^	4
Koga 2021^44^	5^d^	4
Fode 2023^45^	4^d^	4

^a^According to the Newcastle-Ottawa Scale (range, 0-9),^24^ Newcastle-Ottawa Scale adapted for noncomparative studies (range, 0-6),^25^ Jadad Scale (range, 0-5),^23^ or Murad Scale (range, 0-8).^26^;

^b^According to the Oxford Centre for Evidence-Based Medicine 2011 (range, 1-5).^22^

*** Average of the 3 RCTs included in the post hoc analysis.

^c^Newcastle-Ottawa Score.

^d^adapted Newcastle-Ottawa Scale.

^e^Jadad Score.

^f^Average of 3 RCTs included in the post hoc analysis.

^g^Murad Score.

#### CT in ED

**Figure 2:**
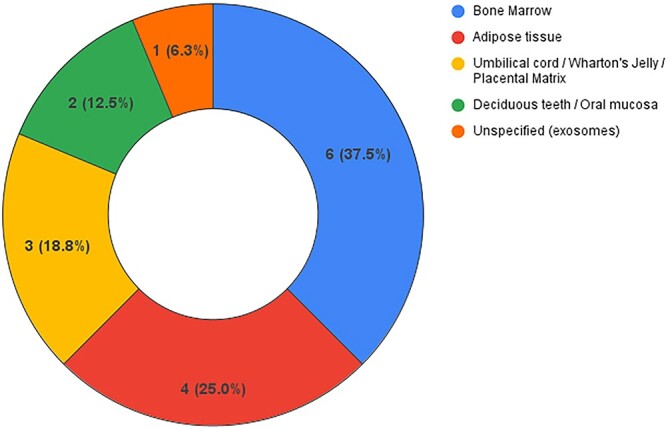
Cell retrieval sites in studies on erectile dysfunction (ED).

Most studies (n = 16, 84%) investigated effects of stem cells in patients with ED.^16^^,^^28^^,^^29^^,^^31^^,^^33-40^^,^^42-45^ A total of 11 papers (69%) included only subjects with ED unresponsive to medical therapies.^16^^,^^28^^,^^29^^,^^33-35^^,^^38-40^^,^^42^^,^^43^ Bone marrow (n = 6, 38%)^28^^,^^33^^,^^35^^,^^37^^,^^39^^,^^42^ and adipose tissue (n = 4, 25%)^29^^,^^34^^,^^36^^,^^45^ were the most common stem cell retrieval sites (Figure 2). The most common validated questionnaire administered to evaluate erectile function was the IIEF or its variations (eg, IIEF-5, IIEF-EF), used in 15 papers (94%),^16^^,^^29^^,^^31^^,^^33-40^^,^^42-45^. Significant improvement in IIEF scores after CT was reportedin 11 of 15 articles (73%).^33-40^^,^^43-45^ More specifically, among studies that showed IIEF score improvement, the mean IIEF-EF score increased between 8 and 14 points,^33^ the mean IIEF-5 score increased between 2 and 9 points,^39^^,^^40^^,^^43^^,^^44^ and the median IIEF-EF score increased between 4.5 and 6 points.^37^^,^^45^

However, only 1 study report included data demonstrating an improvement in IIEF-EF greater than or equal to the minimal clinically important difference (MCID), which was reported in only 33% of patients.^45^ Moreover, in some articles the IIEF improvement only occurred in a specific group of patients (ie, normal erectile function and urinary continence before radical prostatectomy)^34^ or for certain types and quantities of cells (ie, autologous cells, 200 million cells).^37^ One article showed better IIEF-5 scores (>21) after CT in younger patients, men with better pretreatment IIEF-5, and patients with less prevalence of diabetes, hypertension, or priapism.^44^ The included RCTs showed heterogeneous results, with the stuy reports describing some changes in IIEF-5,^16^ significant IIEF-EF improvement only with a high dose of cells,^37^ and significant IIEF-5 improvement in the whole patient cohort,^40^ respectively. All studies for which a significant increase in IIEF was reported (n = 11) showed improvements within 3-6 months.^33-40^^,^^43-45^ Four of 11 (36%) articles described a significant difference from baseline in IIEF still present at 12 months,^33-35^^,^^37^ and 1 of 11 (9%) papers reported a significant reduction in IIEF between 6 and 12 months after initial improvement.^43^ The Erection Hardness Score (EHS) was used in 5 studies (31%), in which patients showed an improvement after CT in all cases (100%).^33-35^^,^^38^^,^^43^ Penile Doppler ultrasound was performed in 7 studies (47%),^31^^,^^36^^,^^38-40^^,^^42^^,^^43^ and in 4 of 7 patients (57%) the peak systolic velocity was improved after CT.^31^^,^^36^^,^^38^^,^^43^

No severe adverse events (AEs) were reported in the articles on ED. In 7 reported studies only mild transient AEs at the donor or recipient site were described (44%),^31^^,^^34-36^^,^^39^^,^^43^^,^^45^ and in a total of 7 papers (44%) no AEs we reported.^16^^,^^28^^,^^29^^,^^33^^,^^40^^,^^42^^,^^44^

The characteristics and main findings of studies on ED are detailed in Table 3. A summary of outcomes of CT in ED patients is reported in Table 4.

**Table 3:** Clinical setting, administered CT, and main findings of studies.

**Reference**	**Clinical setting**	**Type of CT**	**Treatment protocol**	**Efficacy outcomes** ^a^	**Safety outcomes**
Bahk 2010^16^	ED in diabetic patients unresponsive to medical therapies and awaiting penile prosthesis	Allogeneic human umbilical cord blood stem cells	1 injection session of 1.5 × 10^7^ stem cells in both corpora cavernosa. Tourniquet: 30 minutes postinjection Control: normal saline PDE5Is during sexual intercourses	IIEF-5: some changes. Data reported for single patient without summary. Controls: no change. In 6/7 interventional patients within 2 mo morning erections restored for ≥3 mo. Controls: no change (no morning erections) In 6/7 interventional patients: penile hardness improved (still insufficient for effective penetration). Controls: no change In 6/7 interventional patients sexual desire increased in both frequency and intensity Controls: fluctuations occurred. SEP2: at least 1 Yes in 4/7 interventional patients None in controls SEP3: at least 1 Yes in 2/7 interventional patients None in controls GAQ for efficacy of CT to improve erection: with PDE5Is: Yes 5/7 without PDE5Is: Yes 3/7 Testosterone levels: inconsistent changes Diabetic conditions: improved in interventional patients. No improvement in controls	No AEs
Ichim 2013^28^	ED in patients unresponsive to medical therapies and awaiting penile prosthesis	Autologous bone marrow mononuclear cells	60 mL bone marrow aspirate obtained under local anesthesia Bone marrow mononuclear cells concentrated to 2 mL; 1 mL injected in each corpus cavernosum Tourniquet: 5 minutes after injection	At 3 wk: erection strong enough for penetration but inability to maintain erection until orgasm At 3 mo: ability to have intercourse to orgasm and marked increase in morning erections At last visit improved sexual function compared to pretreatment	No AEs
Garber 2015^29^	ED in diabetic patients unresponsive to medical therapies and awaiting penile prosthesis	Autologous adipose stem cells	Liposuction from abdominal subcutaneous fats to obtain stem cells 1 session of injection in both corpora cavernosa of 1.5 × 10^7^ stem cells Tourniquet: 30 minutes after injection PDE5Is during sexual intercourses	IIEF-5: some changes. Data reported for single patient and only for some questions (1,3,4,13) without summary Morning erections: regained in 5/6 patients at 2 mo and maintained for 12 mo Penile hardness: improved in 6/6 patients (still insufficient for effective penetration) Effect of CT on ED: with PDE5Is: Yes 5/6 without PDE5Is: Yes 4/6 (although effect without PDE5Is was insufficient) Sexual desire: increased in both frequency and intensity in 5/6 Testosterone levels: inconsistent changes Diabetic conditions: improved in 6/6 patients	No AEs
Levy 2015^30^	PD patients refusing surgery Penile curvature: 60°-120° in 4 patients, 0° in 1 patient Likely chronic stage, but 1 patient with pain included	Allogeneic placental matrix-derived mesenchymal stem cells from the chorionic placenta	3 mL diluted stem cells solution: up to 2 mL injected in and around plaques, rest injected into both corpora cavernosa	Penile curvature improvement At 6 wk from baseline (4 patients): 10°-120°, 14.9%-100% At 3 mo from baseline (2 patients): 30°-85°, 70.8%-42.8% Plaque volume decreased significantly (as early as 6 wk). 7/10 plaques disappeared completely on ultrasound At 3 mo plaque volume reduction >90% in 5 patients At 6 mo plaque volume reduction close to 100% in 4/4 patients evaluated After injection of trimix solution PSV range improved from 14.1-25.5 (baseline) to 23-42.6 cm/s (6 wk), 38.9-49 cm/s (3 mo), 50.5-67.1 cm/s (6 mo), *P* < .01 No significant change in EDV, IIEF, SPL, penile girth	Priapism: 1 (20%)
Levy 2016^31^	ED in patients who could not tolerate oral therapy and did not want a penile prosthesis.	Allogeneic placental matrix-derived mesenchymal stem cells	1 mL stem cells diluted in 2 mL isotonic saline (number of stem cells in 1 mL not quantified) One session of injection in both corpora cavernosa (1.5 mL diluted solution per corpus) PDE5Is not allowed	After injection of 0.2 mL trimix solution PSV range improved from 23.1-49.3 cm/s (baseline) to 25.5-56.5 cm/s (6 wk), 32.5-66.7 cm/s (3 mo), 50.7-73.9 cm/s (6 mo), *P* < .05 After injection 3 patients were able to achieve erections with no pharmacologic assistance, 4 patients needed low-dose oral medication, and 1 patient continued to use trimix solution No significant change in EDV, IIEF, SPL, penile girth	Transient local irritation: 3 (37.5%)
Lander 2016^32^	Chronic PD patients Penile curvature: mild - 90°	Autologous adult mesenchymal stem cells from stromal vascular fraction	50 cc miniliposuction to obtain stromal vascular fraction under local anesthesia One session of injection in plaque ESWT: 1 treatment before injection, 1 to 6 treatments over next few wk	Subjective reduction in plaque: 11/11 Subjective straightening of penis: 11/11 Improvement of erection: 7/11 (could be related to ESWT) Mean EHS: pre 2.7 vs post 3.5 Mean PDQ: pre 15 vs post 8.7	Minimal abrasion and bruising of penile skin at time of treatment in most of patients
Yiou 2017^33^	Post-RP ED unresponsive to medical therapies	Autologous bone marrow mononuclear cells	Bone marrow aspirates obtained from iliac crests One intracavernous injection, 10^9^ stem cells (stage II) One intracavernous injection with escalating doses (2 × 10^7^, 2 × 10^8^, 1 × 10^9^, 2 × 10^9^) (stage I) Mean time from RP to treatment: 26.3 (stage II) and 24.4 (stage I) mo Medications for ED allowed	Stage II Mean ± SD IIEF-IS: 4.6 ± 2 (baseline) vs 7.2 ± 3.6 (6 mo) *P* = .035; 6.9 ± 3.4 (12 mo) vs 6 ± 3.5 (last follow-up) *P* = .44 IIEF-SD: 6.4 ± 2.7 (baseline) vs 7.6 ± 1.6 (6 mo) *P* = .16; 7.6 ± 1.1 (12 mo) vs 7 ± 1.5 (last follow-up) *P* = .85 IIEF-OS: 3.9 ± 2.3 (baseline) vs 5.8 ± 2.3 (6 mo) *P* = .15; 5.8 ± 2.7 (12 mo) vs 4.4 ± 3.2 (last follow-up) *P* = .14 IIEF-EF (with pharmacotherapy): 7.1 ± 3.1 (baseline) vs 18.4 ± 8.2 (6 mo) *P* = .0091; 18.1 ± 7 (12 mo) vs 15.3 ± 8.1 (last follow-up) *P* = .22 IIEF-OF: 3.8 ± 3.1 (baseline) vs 6.3 ± 2.6 (6 mo) *P* = .024; 6 ± 2.4 (12 mo) vs 5.9 ± 3.8 (last follow-up) *P* = 1 EHS with pharmacotherapy: 1.4 ± .7 (baseline) vs 2.9 ± .8 (6 mo) *P* = .02; 3 ± 0.5 (12 mo) vs 2.5 ± 0.9 (last follow-up) *P* = .11 EHS without pharmacotherapy: 0.6 ± 0.7 (baseline) vs 1.2 ± 1.2 (6 mo) *P* = 0.09; 1.6 ± 1.3 (12 mo) vs 1.3 ± 1 (last follow-up) *P* = .5 Stage I Mean ± SD IIEF-IS: 2.2 ± 3.4 (baseline) vs 7.8 ± 3.1 (at 6 mo); *P* = .033 IIEF-SD: 6.2 ± 1.8 (baseline) vs 6.7 ± 1 (at 6 mo); *P* = .34 IIEF-OS: 3.3 ± 2.4 (baseline) vs 6.8 ± 2.5 at 6 mo); *P* = .035 IIEF-EF (with pharmacotherapy): 3.7 ± 4.1 (baseline) vs 18 ± 8.3 (at 6 mo); *P* = .035 IIEF-OF: 3.3 ± 3.2 (baseline) vs 7.3 ± 2.3 (at 6 mo); *P* = .034 EHS with pharmacotherapy: 1.8 ± .8 (baseline) vs 3.3 ± .8 (at 6 mo); *P* = .053 EHS without pharmacotherapy: .8 ± .8 (baseline) vs 1.2 ± .4 (at 6 mo); *P* = .058	No AEs (stage II) No AEs (stage I) No evidence of PCa recurrence in stage I patients
Haahr 2018^34^	Post-RP ED unresponsive to medical therapies	Autologous adipose-derived regenerative cells	Lipoaspirate of abdominal adipose tissue under general anesthesia to obtain stem cells Mean yield of 1.5 × 10^5^ adipose-derived regenerative cells/g fat tissue 4 mL injected in 2 bilateral points in distal and proximal corpus cavernosum Tourniquet: 30 minutes after injection Mean time between RP and treatment: 10.7 mo Medications for ED allowed	Median (IQR) IIEF-5 6.0 (3) at baseline vs 8 at 12 mo (14); *P* = .004 8 (38%) patients recovered erection sufficient for intercourse: 5 with erectile aids, 3 without erectile aids Improvement in EF (IIEF-5, EHS) was solely demonstrated in patients with normal preoperative EF that were continent at inclusion No significant relationship between nerve-sparing approach and erectile recovery	Transient redness and swelling at injection sites: 8 (38.1%) Reaction in penile area: 3 (14.3%) Minor abdominal hematoma: 5 (23.8%) Abdominal hematoma which led to scrotal and penile hematomas: 1 (4.8%) Light abdominal discomfort after liposuction: 8 (38.1%) Sensitive abdominal skin: 4 (19%) Patient needed analgesic drugs in the days following liposuction: 1 (4.8%)
Al Demour 2018^35^	ED in diabetic patients unresponsive to medical therapies	Autologous bone marrow-derived mesenchymal stem cells	Bone marrow aspirates obtained from iliac crests under local anesthesia 2 sessions of intracavernous injections (at baseline and after 30 days) Four injections per session: one proximal and one distal at each side 30 × 10^6^ cells/4 mL normal saline in 1 mL syringe	Better IIEF-15 at 12 mo compared with baseline. Overall significant difference for total IIEF-15 (*P* = .04), EF (*P* = .03), SD (*P* = .04), IS (*P* = .04), OS (.04), OF (>.05) EHS: range 0-1 at baseline, range 1-4 at 12 mo; at 12 mo improved from baseline in all patients (*P* = .02)	Pain during BM aspiration procedure: VAS range 2-4 No significant AEs
Protogerou 2019^36^	ED	Autologous adipose derived mesenchymal stem cells	Group A: CT + platelet lysate Group B: platelet lysate Lipoaspiration to obtain stem cells. Group A received 38.9 x 10^6^ stem cells combined with 2.2 mL platelet lysate Medication for ED allowed One intracavernous injection Tourniquet: 10 minutes	Group A: -IIEF-5: range 6-16 (baseline), range 6-22 (3 mo). Improvement of IIEF-5 in 4/5 patients. IIEF-5 baseline vs 3 mo improved (*P* < .05) -PSV: range 16.1-45.5 cm/s (baseline), range 39-97.9 cm/s (3 mo), improvement of PSV in 4/5 patients -Reappearance of morning erections -Reducing need for ED medications No statistically significant difference in IIEF-5 between Group A and Group B	Minor pain at time of injection in all patients, but less intense in Group A
Ory 2020^37^	ED in patients with ischemic cardiomyopathy	POSEIDON: Autologous vs allogeneic mesenchymal stem cells derived from bone marrow TAC-HFT: Autologous bone marrow-derived mesenchymal stem cells vs autologous bone marrow mononuclear cells TRIDENT: Allogeneic bone marrow-derived mesenchymal stem cells	Transendocardial injection of stem cells or placebo via cardiac catheterization. POSEIDON: 20, 100, 200 million cells TAC-HFT: 200 million cells TRIDENT: 20, 100 million cells 9 received 20 million cells, 8 received 100 million cells, 11 received 200 million cells	Median (IQR) IIEF-EF at baseline: 5.5 (1.5-8.5) placebo group vs 5 (1.3-14) CT group; *P* = .878 Median (IQR) IIEF-EF at 12 mo: 3.5 (3-5.8) placebo group vs 7 (1.5-20) CT group; *P* = .486 Not significant IIEF-EF change from baseline to 12 mo in placebo group (*P* > .05) Not significant IIEF-EF change from baseline to 12 mo in CT group (*P* > .05) Significant improvement of median (IQR) IIEF-EF from baseline to 12 mo in men who received 200 million cells: 14 (4-23) vs 20 (15-24.5), *P* = .014 Significant improvement of median (IQR) IIEF-EF from baseline to 12 mo in men who received autologous cells: 14 (3.8-23.3) vs 20 (12-22), *P* = .030	NA
Zasieda 2020^38^	Severe organic ED in patients with metabolic syndrome and generalized atherosclerosis and poor response to PDE5Is	Mesenchymal stem cell-derived exosomes	Group A: CT + ESWT Group B: ESWT Both groups also took 50 mg daily of Ikariin +1.0 g of L-arginine aspartate Group A: 6 wk combined treatment with 6 sessions of intracavernous mesenchymal stem cell-derived exosomes (one a week) + 12 sessions of ESWT (twice a week). Injections were provided 30 minutes after ESWT by injecting 2.5 mL of solution intracavernously in each peduncle of penis (total solution injected: 5.0 mL)	In Group A statistically significant improvement of IIEF-5, EHS, PSV, and EDV. Pre vs post treatment number of patients with: -mild–moderate ED: 0 vs 6 (*P* < .01) -moderate ED: 0 vs 12 (*P* < .01) -severe ED: 19 vs 0 (*P* < .01) -EHS Grade 3: 2 vs 9 (*P* < .01) -EHS Grade 4: 17 vs 2 (*P* < .01) -PSV: 23.4 ± 0.3 vs 29.6 ± 0.3 (*P* < .01) -EDV: 5.4 ± 0.2 vs 4.5 ± 0.3 (*P* < .01) Comparison of posttherapeutic IIEF-5, EHS, PSV, and EDV between groups showed absence of significant differences except for PSV in favor of Group A (29.6 ± 0.3 cm/s vs 25.4 ± 0.3 cm/s, *P* < .01)	NA
Bieri 2020^39^	Vascular ED refractory to PDE5Is	Autologous bone marrow concentrate	Clinical trial Bone marrow aspiration in local anesthesia: 30 mL (low dose) or 60 mL (high dose) per patient Extraction of 3 mL (low dose) or 6 mL (high) of bone marrow concentrate. 1.7 × 10^8^ cells concentrated into 3 mL Injection into both corpora cavernosum along dorso-lateral aspect of proximal third of penis of 1.5 mL (low dose) or 3 (high dose) mL bone marrow concentrate Registry Injection of 10 mL bone marrow aspirate into each corporal body Tourniquet: 15 minutes	IIEF-5 in Low dose group Mean (range) Baseline: 8 (5-16) 6 mo: 10 (5-22) IIEF-5 in High dose group Mean (range) Baseline: 9 (5-16) 6 mo: 12 (5-20) IIEF-5 in Registry Mean (range) Baseline: 9 (5-15) 6 mo: 18 (10-23) *P* = .001 No statically significant change in doppler ultrasound and cavernosmetry in low or high dose group (*P* > 0.05)	Clinical trial Low dose group Pain at harvest site: 5 (25%) Pain at injection site: 6 (30%) Brushing at harvest site: 5 (25%) Bruising at injection site: 3 (15%) High dose group Pain at harvest site: 7 (35%) Pain at injection site: 6 (30%) Brushing at harvest site: 5 (25%) Bruising at injection site: 4 (20%) Registry Pain at harvest site: 9 (9%) Pain at injection site: 5 (5%) Brushing at harvest site: 6 (6%) Bruising at injection site: 4 (4%)
Mirzaei 2021^40^	ED in diabetic patients unresponsive to common therapies	Autologous mesenchymal stem cells from oral mucosa	After local anesthesia sampling of 0.5 cm of oral mucosa. In control group only insertion of a swap into patient’s mouth to simulate sampling 50-60 × 10^6^ stem cells diluted in normal saline (up to 2 mL) and injected into corpora cavernosa (1 mL per each corpus). In control group injection of normal saline into corpus cavernosum. PDE5Is during sexual intercourses	Mean IIEF-5 in CT group 7.2 ± 2.1 (baseline) vs 10.6 ± 4.7 (6 mo; *P* = 0.01 Mean IIEF-5 in control group 7.2 ± 2.1 (baseline) vs 7.3 ± 2.1 (6 mo); *P* = 0.87 Mean IIEF-5 in CT group vs Mean IIEF-5 in control group at sixth mo significantly in favor of CT (*P* = .02) No statistically significant difference between baseline and 6 mo in both groups for PSV, EDV, and RI (*P* > .05) No statistically significant difference between groups for PSV (*P* = .25), EDV (*P* = 1), and RI (*P* = .057) Morning erection recovered in 2 (CT) vs 0 patients (control group)	None of patients in neither control nor intervention group reported injection-related complications (bleeding, hematoma, ecchymosis, abscess, etc.) after six mo follow-up
Nguyen Thanh 2021^41^	Males with reduced sexual desire and testosterone levels ≤12 nMol/dL	Autologous adipose-derived mesenchymal stem cells	Adipose tissue harvested from lower abdomen under general anesthesia Stem cells resuspended in 20 mL normal saline 1x10^6^ cells/kg body weight infused through intravenous route	Mean (SD) IIEF-EF: 19.0 (7.82) at baseline vs 23.7 (5.16) at 12 mo (*P* < .05) Mean (SD) IIEF-OF: 7.47 (2.26) at baseline vs 7.73 (2.12) at 12 mo (*P* > .05) Mean (SD) IIEF-SD: 6.07 (2.05) at baseline vs 6.60 (1.64) at 12 mo (*P* > .05) Mean (SD) IIEF-IS: 7.33 (3.58) at baseline vs 9.33 (2.41) at 12 mo (*P* < .05) Mean (SD) IIEF-OS: 5.53 (1.36) at baseline vs 7.07 (1.33) at 12 mo (*P* < .05) Mean (SD) testosterone levels: 9.99 (3.13) nMol/dL at baseline vs 11.8 (4.27) nMol/dL at 12 mo (*P* < .05)	Only nonserious AEs related to CT (impossible to discriminate AEs in subgroup of men)
You 2021^42^	ED in patients unresponsive to PDE5Is (5 with post-RP ED, 5 with diabetes-associated ED)	Autologous bone marrow-derived mesenchymal stem cells	10 mL bone marrow obtained from superior iliac crest under local anesthesia 3 x 10^7^ stem cells suspended with 2 mL plasma solution One injection into corpus cavernosum (on right or left side) Tourniquet: maintained for 30 minutes PDE5Is allowed	No statistically significant difference from baseline and 12 mo in IIEF, SEP2, SEP3, PSV, and RI Mean IIEF increased significantly at 1 month vs baseline (24.9 vs 18.1, *P* = .0222)	No AEs related to CT
Al Demour 2021^43^	ED in diabetic patients unresponsive to medical therapies	Allogeneic Wharton’s Jelly-derived mesenchymal stem cells	Wharton’s Jelly collected from O Rh-negative healthy donors, full-term women, who underwent elective cesarean section 20 × 10^6^ cells/4 mL normal saline loaded into 1 mL sterile syringes 2 sessions of intracavernous injections with a 30-day interval Each session consisted in 4 injections: 1 proximal and 1 distal in into each corpus cavernosum. ED medications not allowed	Mean (SD) IIEF-5 11.5 ± 2.7 (baseline) vs 16.9 ± 4 (6 mo) *P* < .0001 Mean (SD) IIEF-5 16.9 ± 4 (6 mo) vs 13.6 ± 4.2 (12 mo) *P* = .0002 Mean EHS (SD) 1.7 ± 0.7 (baseline) vs 3 ± 0.8 (6 mo) *P* < .0001 Mean EHS (SD) 3 ± 0.8 (6 mo) vs 2.4 ± .7 (12 mo) *P* < .0001 Mean basal PSV (SD) 12.06 ± 10.33 (baseline) vs 16.35 ± 12.71 (3 mo) *P* = .0332 Mean 20-min PSV (SD) 38.34 ± 12.93 (baseline) vs 48.72 ± 17.05 (3 mo) *P* < .0001 No significant difference in basal EDV, 20-min EDV, basal RI, and 20-min RI between baseline and 3 mo	Mild pain at injection site only during procedure (VAS pain 0-3): 10 (45.5%) Minimal redness and swelling at base of penis and bruises at distal shaft of penis 24 hour after first injection: 2 (9.1%) Very small fibrous plaque (PD) on dorsal aspect of penis without curvature, 3 mo after second injection, which did not interfere with sexual intercourse: 1 (4.5%)
Koga 2021^44^	ED	Allogeneic stem cells from human exfoliated deciduous teeth	The majority of patients 3 injection sessions at a week interval (32 patients 3 sessions, 3 patients 5 sessions, 2 patients 8 sessions, 1 patient 1 session) Each injection session: injection of 2 cc stem cells into both corpora cavernosa Hairband loosely attached to base of penis for 6 hours ED medications not allowed	Mean (range) IIEF-5: 13.1 (5-20) at baseline vs 19.3 (7-25) after 3 injection sessions, *P* < .0001 Significant increase in IIEF-5 between each injection until third injection Better IIEF-5 scores in patients younger, with better pretreatment IIEF-5, and with less prevalence of diabetes, hypertension or priapism No significant change in testosterone levels	No AEs
Fode 2023^45^	Vasculogenic ED	Autologous adipose-derived stem cells	50-60 mL lipoaspirate Single-site intracavernous injection of 4 mL stem cells Tourniquet: no ED medications not allowed	Median IIEF-EF: 5.5 (baseline) vs 10 (3 mo) *P* < .0078 No significant difference in median IIEF-OF, IIEF-SD, IIEF-IS, and IIEF-OS between baseline and 1, 2, 3 mo (except for IIEF-OS at 2 mo) 3 (33.3%) patients achieved improvement equal to or ≥MICD according to their baseline IIEF-EF	Minor discomfort for fat harvest and penile injection Minor blue discoloration at fat harvest site

Abbreviations: AE, adverse event; CT, cell therapy; ED, erectile dysfunction; EDV, end-diastolic velocity; EHS, Erection Hardness Score; ESWT, extracorporeal shock wave therapy; GAQ, global assessment questions; IIEF, International Index of Erectile Function; IIEF-5, IIEF 5-item version; IIEF-EF, IIEF-Erectile Function; IIEF-IS, IIEF-Intercourse Satisfaction; IIEF-OF, IIEFOrgasmic Function; IIEF-OS, IIEF-Overall Satisfaction; IIEF-SD, IIEFSexual Desire; IQR, interquartile range; NA, not available; PCa, prostate cancer; PD, Peyronie’s disease; PDE5Is, phosphodiesterase 5 inhibitors; PDQ, Peyronie’s Disease Questionnaire; PSV, peak systolic velocity; RI, resistance index; RP, radical prostatectomy; SEP, sexual encounter profile; SPL, stretched penile length.

^a^Referred to last follow-up visit if not specified.

**Table 4:** Summary of outcomes of CT in ED patients.

**Efficacy outcomes**	**Safety outcomes**
IIEF ^a^significant improvement: 11/15 studies (73%) IIEF-5 mean increase: 2-9 points IIEF-EF median increase: 4.5-6 points IIEF-EF improvement ≥MCID: 33% of patients IIEF* improvement only/superior in selected groups of patients (eg, normal EF before RP, higher dose of cells, younger men, better pretreatment EF, fewer comorbidities) Heterogeneous/contradictory effects on IIEF^a^ considering only RCTs EHS improvement: 5/5 studies (100%) PSV improvement: 4/7 studies (57%)	Mild transient AEs at donor or recipient site: 7 studies (44%) No severe AEs No AEs: 7 studies (44%) NA: 2 studies (13%)

Abbreviations: AE, adverse event; CT, cell therapy; ED, erectile dysfunction; EF, erectile function; EHS, Erection Hardness Score; IIEF, International Index of Erectile Function; IIEF 5-item version; IIEF-EF, IIEFErectile Function; MCID, minimal clinically important difference; NA, not available; psv, peak systolic velocity; rct, randomized controlled trial; RP, radical prostatectomy.

^a^IIEF-15 or its variations (IIEF-5 or IIEF-EF).

#### CT in other MSD

A minority of studies (n = 3, 16%) evaluated the impact of CT on MSD effects other than ED.^30^^,^^32^^,^^41^ More specifically, 2 reported studies (11%) were focused on PD,^30^^,^^32^ and 1 reported study (5%) investigated male patients with reduced sexual desire and testosterone levels.^41^ The main findings of the articles mentioned in this section are described in Table 3.

### CT in PD

The 2 studies on PD included in this review investigated the effects of injection into the plaques of allogeneic placental matrix–derived mesenchymal stem cells^30^ and autologous mesenchymal stem cells from the stromal vascular fraction.^32^ Enrolled patients were in the chronic phase, with curvature between mild and 120°. In both articles, penile curvature improvement and plaque volume reduction were described in all patients (100%).^30^^,^^32^ However, objective measurements were performed in only 1 study, which showed 10°-120° (15%-100%) curvature improvement at 6 weeks and 90%-100% plaque reduction.^30^ A reduction in the PD Questionnaire (PDQ) score was reported in the other study.^32^ No severe AEs were recorded. One case of priapism (1of 5 patients, 20%)^30^ and mild penile skin complications in the majority of patients^32^ were reported among the 2 studies.

### CT in low sexual desire and testosterone levels

The articles on men with low sexual desire and testosterone levels described the effects of autologous adipose-derived mesenchymal stem cells infused through the intravenous route.^41^ At 12 months from baseline, statistically significant improvements in IIEF-EF and testosterone levels were reported; however, no increase was found scores for the IIEF-Sexual Desire questionairre. The authors recorded only nonserious AEs related to CT. In 3 articles on ED, testosterone levels were not reported to have changed significantly.^16^^,^^29^^,^^44^ Sexual desire was reported to have increased in some studies of ED patients^16^^,^^29^^,^^35^; however, in other studies it did not change.^33^^,^^45^

## Discussion

This investigation is to our knowledge the most comprehensive and up-to-date systematic review thus far evaluating the use of CT in managing MSD. This study highlights the potential benefits and limitations of CT treatment in male sexual medicine and the characteristics of the literature available on the topic. Furthermore, the use of validated tools and a panel of experts has allowed the formulation of official ESSM position statements.

Present data suggest a possible improvement of erectile function after CT; however, several considerations are necessary in this regard, and the available data should be interpreted with extreme caution. First, 2 of the 3 included RCTs did not find a statistically significant difference in IIEF scores between the examined groups.^16^^,^^37^ In addition, in a conference abstract, Hansen et al.^46^ have recently presented the results of a randomized double-blind placebo-controlled phase 2 trial. Interestingly, this trial was an extension of the single-arm phase 1 study by Haahr et al.^34^ This new RCT showed no statistical difference between groups in IIEF-5 and EHS at 1, 3, 6, and 12 months, contradicting the preliminary findings of the previous study. Hence, the vast majority of the available RCTs on the topic report discouraging results, highlighting the possibly that the positive findings in most other papers could simply be attributable to their low quality.

Moreover, the magnitude of the observed effects varied over a wide range and was almost never adequately investigated. Only 1 study reported MCID scores for the IIEF-EF (2, 5, and 7 for patients with mild, moderate, and severe baseline ED, respectively),^47^ showing an improvement greater than or equal to othe MCID in only 33% of cases.^45^

Limited evidence indicates that proper patient selection could be critical for the efficacy of CT in ED patients. Indeed, better outcomes were found in men with greater erectile function before cell administration, normal erectile function and urinary continence before radical prostatectomy, younger age, and lower prevalence of some comorbidities.^34^^,^^44^ The cell dose may also influence the efficacy of CT for ED; however, the included articles appear contradictory on this point.^37^^,^^39^

Most studies enrolled patients unresponsive to conservative ED therapies.^16^^,^^28^^,^^29^^,^^33-35^^,^^38-40^^,^^42^^,^^43^ This outcome is reasonable owing to the experimental nature of CT, due to which it was not proposed as a first line of treatment. On the other hand, this characteristic of the enrolled patients allows us to hypothesize that the selected patients were the most “difficult” to treat; consequently, the efficacy of CT may have been underestimated. In this context, it is important to underline that about half of the reported studies allowed or encouraged the concomitant use of ED medications with CT,^16^^,^^29^^,^^33^^,^^34^^,^^36^^,^^40^^,^^42^ assuming a synergistic action between the treatments. According to reported details, several studies showed greater efficacy of CT when associated with pharmacotherapy,^16^^,^^29^^,^^33^^,^^34^ and Protogerou et al. reported a reduction in the need for ED medication in patients undergoing CT.^36^

In the 2 articles reporting studies in which CT alone was compared to the combination of CT with another regenerative therapy (ie, shockwaves or platelet lysate), a statistically significant improvement of IIEF-5 was found in both groups, with no significant difference between the groups.^36^^,^^38^ Therefore, the lack of a synergistic effect with other regenerative treatments can be speculated, but there are insufficient data to draw conclusions on the efficacy of CT compared to the other regenerative options.

Finally, some considerations should be addressed with regard to the timing of onset and the duration of effect after the administration of CT. Several studies reported improvement in IIEF starting 3-6 months after treatment^33-40^^,^^43-45^; this latency period is reasonable given the regenerative nature of the therapy. However, a not negligible number of reported studies showed improvement in erectile function within the first month,^16^^,^^28^^,^^29^^,^^35^^,^^36^^,^^42-45^ suggesting that more immediate mechanisms may exist and that some studies may have found effects at 3 months just because that was the first scheduled posttreatment evaluation. This hypothesis remains controversial since some articles reported significant improvement after 3-6 months but not at 1 month.^33^^,^^34^ On the other hand, some studies demonstrated a persistent beneficial effect on erectile function that lasted up to 12 months^33-35^^,^^37^; conversely, a another reported study found worsening of erection between 6 and 12 months after the initial improvement.^43^ Such findings indicate that CT may be able to regenerate penile tissues but certainly cannot cure all causes of ED; these underlying causes can override the beneficial effect of the treatment over time as they continue to damage the tissues. Clear conclusions on the duration of the effect of CT cannot be drawn due to the lack of long-term studies; however, the probable temporary nature of benefits induced by CT is a fundamental point to take into account, as patients undergoing regenerative therapies are typically looking for a definitive solution.^43^

In ED patients, CT would seem safe; indeed, no severe AEs were recorded in the studies evaluated. Only mild local complications occurred at the donor and recipient sites.^31^^,^^34-36^^,^^39^^,^^43^^,^^45^ However, it is essential to point out that small samples size of included articles and lack of long-term data prevent the drawing of conclusions on uncommon side effects and possible late complications (including cancer risk).

Surgery remains the therapy of choice in men with chronic PD who require active therapy for penile curvature. Nevertheless, if patients desire a noninvasive approach, intralesional treatment with collagenase *Clostridium histolyticum* or interferon-α2b may be an option.^17^ Since *Clostridium histolyticum* was withdrawn from the European market^48^ and the use of interferon-α2b was associated with multiple AEs and high costs,^49^ other substances are under investigation for intralesional therapy, including CT, platelet-rich plasma, and hyaluronic acid.^50^

Interesting preliminary data were found regarding the application of CT in chronic PD. The mechanism of action of CT in this clinical setting remains unclear. Preclinical studies demonstrated the antifibrotic activity of stem cells. More specifically, they appear to be able to decrease collagen deposition, reduce the number of myofibroblasts, diminish the expression of tissue inhibitors of metalloproteinases, enhance the expression of matrix metalloproteinase, and inhibit several fibrosis-related cellular signaling pathways.^15^^,^^51^

Both clinical studies on PD that we investigated showed significant plaque size reduction and penile curvature improvement in all patients, in some cases with complete resolution.^30^^,^^32^ However, the data supporting such apparently promising results derive from single-arm studies characterized by low quality and high risk of bias, which need to be confirmed in robust RCTs. Only 1 case of a patient with priapism and mild local AEs was recorded after CT for PD,^30^^,^^32^ but again, the reported safety data for this treatment are currently very limited and need to be confirmed with adequate RCTs.

Interestingly, 1 article on PD reported an enrolled apatient with penile pain without curvature.^30^ The outcomes of this patient were not described in the paper, but this case suggests a possible use even in the acute phase of the disease. Stem cells have an anti-inflammatory and antifibrotic effect^14^; therefore, CT in acute PD could reduce pain and prevent/attenuate fibrosis. However, this conclusion remains a speculation that needs to be confirmed with appropriate clinical trials.

The impact of CT on sexual desire and testosterone levels was specifically investigated by only one study,^41^ while other papers reported only scattered data in this regard.^16^^,^^29^^,^^33^^,^^35^^,^^44^^,^^45^ The results on the topic are contradictory and the evidence is too low to draw any kind of conclusions.

Despite the results obtained, the data reported here should be read and interpreted with the consideration of several limitations. First of all, the studies included are relatively few and overall have a small sample size, short follow-up, and uncontrolled design, showing a low quality. On the other hand, the heterogeneity of cells used; preparation methods, doses, administration protocols; and tools to evaluate the outcomes make it difficult to compare different studies and draw general conclusions. All of the above factors prevent the performance of a meta-analysis (excluded a priori) and affect the formulation of position statements. Furthermore, it should be considered that many of the available studies were designed to evaluate the feasibility or safety of CT as the primary outcome; this approach limits the reliability of the efficacy data. Finally, according to the details reported on ClinicalTrials.gov, it is possible to hypothesize that the available evidence suffers from significant bias resulting from the suspension of several studies due to lack of funding, recruitment difficulties or poor efficacy, and consequent nonpublication of related data.

A particular effort should be made to develop well-designed RCTs on CT for MSD. Placebo-arm, blinding, large sample size, and extended follow-up are essential characteristics for future studies to offer an adequate LoE. Another fundamental point is to evaluate sexual outcomes only with validated and commonly used questionnaires to facilitate comparability of study results. The magnitude of the effects should be explored appropriately to understand if they are clinically significant. Preparation methods, doses, and administration protocols of CT should be standardized to make comparisons between different articles more reliable. Comparative studies between different types of cells should be developed. Long-term side effects (including the risk of cancer, especially in patients with a personal history of previous tumors), time required for the onset of the effect, and duration of any benefit obtained should be evaluated adequately in future papers. Research to determine the predictors of better outcomes after CT should be planned to facilitate the choice of the best candidates for this treatment. Finally, future studies on CT should also investigate other fields of male sexual health that are partially or totally unexplored, such as PD, premature ejaculation, orgasmic dysfunctions, and sexual desire disorders.

Unfortunately, the future of ongoing CT research currently does not look bright. High research costs, difficulty obtaining approval from local Ethics Committees, and legal issues to patent CT technology are significant obstacles. These factors may explain why research on the topic is progressing so slowly in the last decades and will likely prevent many high-quality studies from being conducted in the coming years.

In conclusion, preliminary findings are available in favor of efficacy and safety of CT in patients with ED or PD, suggesting a potential application of CT in these patients. However, the supporting evidence is very limited, due to low-quality papers, consistent methodological heterogeneity, uncertainty about the magnitude of the supposed beneficial effects, and lack of long-term data. Consequently, CT should be considered a treatment under investigation and offered only within clinical trials. Further research is needed to improve the knowledge, standardize the treatment, formulate strong recommendations based on high-quality evidence, and ultimately implement CT in regular clinical practice.

## Supplementary Material

Supplementary_Figure_1_qfad071

## Data Availability

The protocol was registered in the International Prospective Register of Systematic Reviews (PROSPERO) database with the ID CRD42023404234.
