# Supplementary figures and images for: Cell therapy for male sexual dysfunctions: systematic review and position statements from the European Society for Sexual Medicine
Source: Sex Med. 2024 Feb 9;12(1):qfad071. doi: 10.1093/sexmed/qfad071 (PMC10857898; doi:10.1093/sexmed/qfad071)

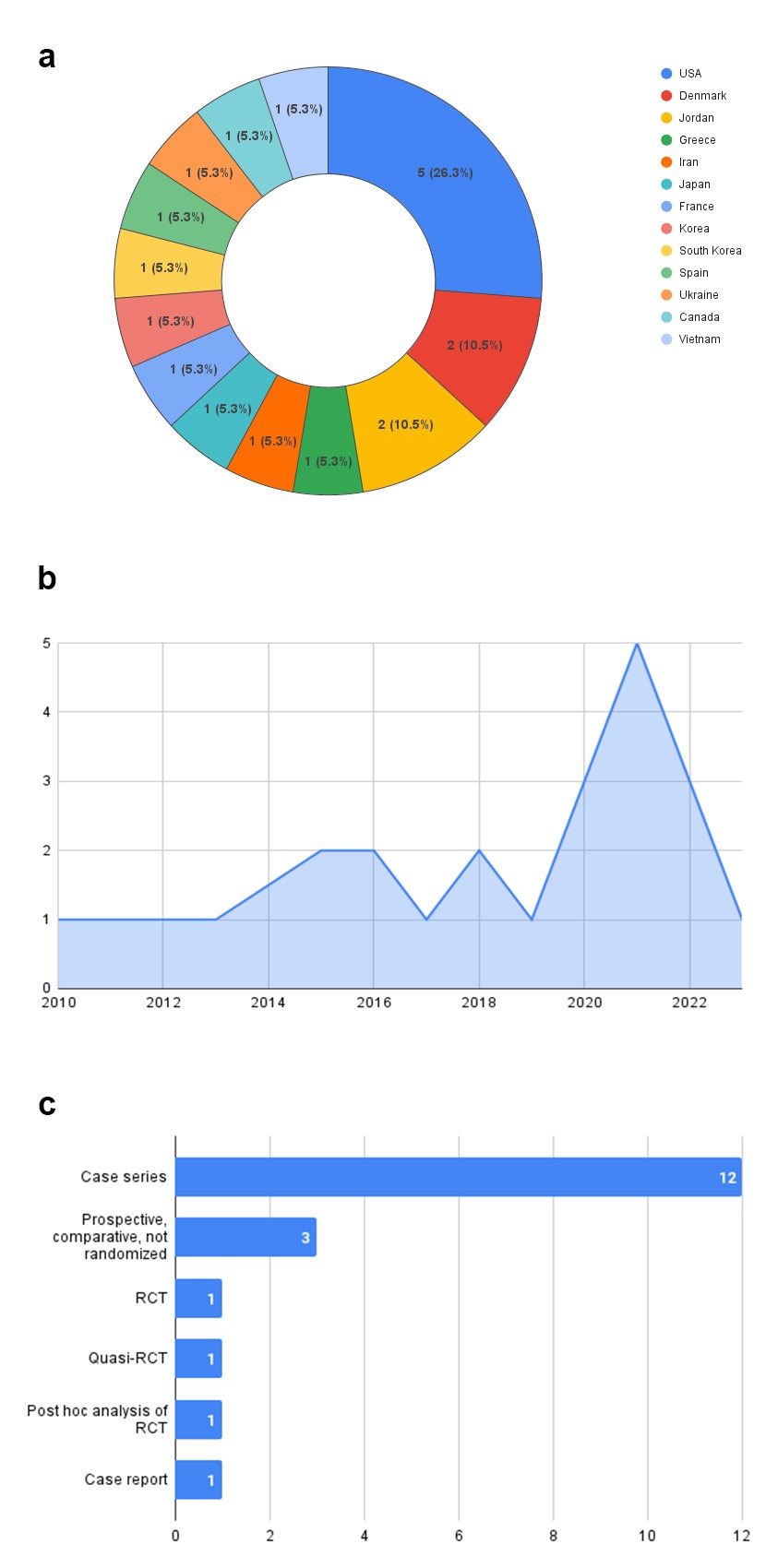

Supplement: Supplementary_Figure_1_qfad071 [file supplementary_figure_1_qfad071.jpeg]
